# The peripheral and Central Humphrey visual field – morphological changes during aging

**DOI:** 10.1186/s12886-017-0522-3

**Published:** 2017-07-17

**Authors:** Paul Rutkowski, Christian Albrecht May

**Affiliations:** 1 282 Harison Ave., Harrison, NY USA; 2 Department of Anatomy, TU Dresden, Dresden, Germany; 3 Anatomisches Institut, Fetscherstr. 74, 01307 Dresden, Germany

**Keywords:** Central visual field, Peripheral visual field, Healthy, Aging

## Abstract

**Background:**

To define age-related changes in the visual field by comparing ‘standard’ central and unique peripheral visual field measurements in healthy volunteers.

**Methods:**

In a single center, retrospective, Cross-sectional, observational study, 20 volunteers with no retinal diseases or risk factors, ranging in age between 30 and 94 years (four age groups: 30’s, 50’s, 70’s, 90’s) were measured in one eye (preferentially the right one) using a Humphrey visual field 24–2 and 60–4.

**Results:**

While the central visual field remained relatively well preserved during aging showing only a mild reduction in sensitivity, a profound loss of the peripheral visual field was observed beginning in the fifth decade of life and decreasing continuously up to the 90ies.

**Conclusions:**

The peripheral visual field declined substantially from the 4th decade onward while the central visual field remained quite stable.

**Electronic supplementary material:**

The online version of this article (doi:10.1186/s12886-017-0522-3) contains supplementary material, which is available to authorized users.

## Background

It is a frequent clinical experience that visual acuity can be maintained with advancing age although visual impairment may increases during this period of one’s life [1]. Particularly advancing age may show difficulty in vision-related actions affecting daily life like driving and even walking when no ocular diseases exist [2, 3]. This dilemma raises two basic questions. Are there any aging processes affecting the inner retina that can help explain this contradiction of vision-related impairment with continued normal visual acuity? If so, do they affect both the central and peripheral retina equally and simultaneously?

Tissue samples of the human inner retina concerning aging are rare, because it is difficult to obtain appropriate tissue and the necessary numbers of tissue samples especially from young donors. To date the four studies found in the literature consist of twelve eyes, divided into a younger (<40 years) and an older (>60 years) age group. All studies revealed a reduction of neurons in the retinal ganglion cell layer [4–7].

An indirect method of estimating the number of retinal ganglion cells loss during aging is to measure the loss of the retinal ganglion axons in the optic nerve over time. In the first study examining 300 optic nerves (age 1 to 96) the results implied an influence of age on the loss of neurons in the optic nerve [8]. Age-dependent changes were subsequently described on smaller samples estimating an annual loss of 4000–7200 axons [9–12]. In contrast, Repka and Quigley reported no inter-individual variation of the axon numbers with age. However there sample was small and 4 out of 5 specimens were from subjects between 60 and 75 years of age [13].

Due to the introduction of new optic systems in the 1990s, mainly the Heidelberg Retina Tomography, the Nerve Fiber Analyzer and the Optical Coherence Tomography, numerous clinical cross sectional studies described thickness variations of the peripapillary retinal nerve fiber layer between healthy young and elderly subjects (Additional file 1: Table S1). Although numerous authors calculated a constant age-dependent regression coefficient through their samples, only two studies reported an age effect beginning at the 6th decade of life [14, 15]. The individual variations of the retinal nerve fiber layer with age (longitudinal studies 2.5 to 3 years) were performed by two groups with contradictory results: in one study laser scanning polarimetry showed a tendency of decreased retinal nerve fiber layer thickness while optical coherence tomography detected an increase in retinal nerve fiber layer thickness [16]. Another study using optical coherence tomography detected no changes with age in the nasal and temporal quadrants but a mean decrease of retinal nerve fiber layer thickness of 1.25–1.35 μm/ year in the superior and inferior quadrants [17].

Summarizing both the histological and clinical data aging appears to have an effect on the retinal ganglion cells, retinal ganglion cells axons and probably the nerve fiber layer. Unfortunately all these studies focus on the central retina at the posterior pole of the eye and the optic nerve head, but none on the distal peripheral retina.

We have attempted to answer some of these aging questions by examining the retinal sensitivity of both the central and peripheral retina using the Humphrey Visual Field (HVF) Ananalyzer specifically the threshold test 24–2 and 60–4. The clinically observed variations of these visual field sensitivity measurements were correlated to the different vascular supply of the retina ganglion cells described previously [18]. The HVF 60–4 threshold test had to be modified because one’s normal nasal retina extends beyond 60 degrees to 80 degrees however the HVF 60–4 extends nasally to only 60 degrees.

**Fig. 1:**
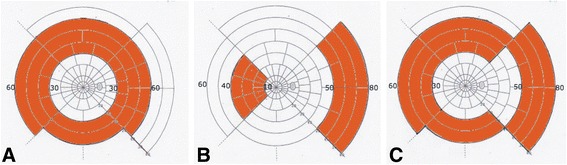
Schematic drawing of a modified Goldmann Overlay Map showing the retinal ganglion cell density in a right eye visual field projection (see Rutkowski and May 2016). **a** The area measured with an ordinary 60–4 Humphrey visual field adjustment is marked in colour; note that the temporal measurements reach only 60 degree. **b** The area measured with a 20 degree nasal to the fovea adjustment is marked for the nasal (*left*) and temporal (*right*) region; note that now the measurement covers up to 80 degree in the temporal side. **c** The collage demonstrates the combination of both measurements evaluated

With this modified HVF 60–4 referred to as (collage) one can measure the effect of aging on both the temporal retina extending from 30 to 60 degrees but also the peripheral nasal retina extending from 50 to 80 degrees [18]. The retinal equator is not only the separation between the anterior and posterior choroidal circulation but also the beginning of the retinal Ora Watershed Zone that occurs between 43 and 63 degrees in the temporal retina and 61 and 80 degrees in the nasal retina. (Fig. 1a). In order to correct for this nasal watershed zone the modified HVF 60–4 was created by moving fixation 20 degrees nasally to standard foveal fixation (Fig. 1b). It was then combined with the standard HVF 60–4 to create a collage (Fig. 1c).

## Methods

Twenty healthy non-smoking volunteers ranging in age from 30 to 94 years without a history of diseases affecting the retina (e.g. diabetes mellitus, hypertension, amblyopia), with no apparent neurological deficits (clinical investigation), and without peculiarities in a standard Heidelberg retinal tomography (HRT, Heidelberg Engineering GmbH, Heidelberg, Germany) were examined. A positive ethics approval and consent was given from the Ethikkommission der TU Dresden (reference number EK 428102016) and adhered to the tenets of the Declaration of Helsinki. A written informed consent for participation in the study and for publication of anonymized images was obtained from all participants. A copy of the written consent is available for review by the Editor of this journal. The volunteers were grouped in four decades: 30–40, 50–60, 70–80 and over 90 years of age.

Prior to testing, one drop of tropicamide 1% was installed in the eye to be tested. A complete eye examination was performed which included gonioscopy, pachymetry, and tonometry, to determine if there was any ocular disease. A Carl Zeiss Humphrey 750 Field Analyzer using the Sita fast technique was performed in a separate dark quiet room.. The HVF 24–2 was performed first, followed by a HVF 60–4 which was repeated with fixation moved 20 degrees nasaly. The duration of the HVF 24–2 was 4–6 min and the HVF 60–4 was 6–10 min (individual duration and number of errors is listed in Additional file 2: Table S2). The reliability of the HVF decreased considerably after age 80 requiring each subject to repeat the three different HVF tests several times. For analysis, the test with the lowest number of fixation loss and errors was used. (single measurement data provided as additional file 3).

Both the HVF 60–4 and the HVF 60–4 collage used the HVF 60–4 program of the Zeiss instrument with fixation moved nasally by attaching a red spot 80 mm nasal to standard fixation. The greater deviation from standard threshold depth measured in decibels the change in the printout from gray to black.

No standardized visual field indices exist for the 60–4 program today. We used the sum score of the visual field threshold of the four quadrants superior-nasal, superior-temporal, inferior-temporal, and inferior-nasal to compare the individual measurements within and between the age groups. Since each quadrant has individual counts of single thresholds they could not statistically compared with each other.

Using a public calculation tool (powerandsamplesize.com) the sample size was tested with a power of α = 0.01. The minimal number for the expected changes was *n* = 14. Since we used more than two age groups we extended the number to *n* = 20. We performed descriptive statistics and included the U-test as the most basic testing with the least requirements (*p* < 0.05 is considered as significant). The evaluation was limited to a pure comparison of the single quadrants of the different age groups since advanced statistic (e.g. regression analysis) requires higher numbers of cases or equal quadrant settings not including all data obtained.

## Results

**Fig. 2:**
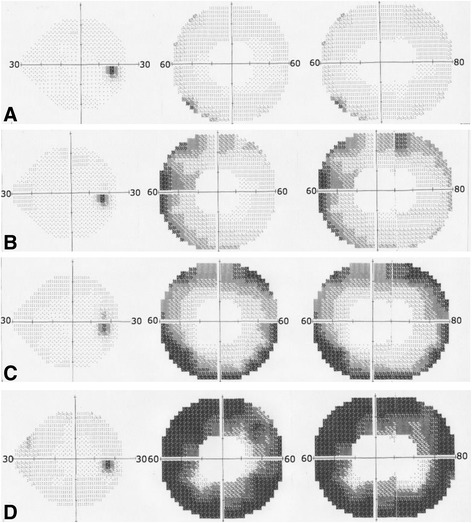
Examples of the different visual fields (left 24–2; middle 60–4; right 60–4 collage) of each age group: **a** a 32 years old female, (**b**) a 56 year old male, (**c**) a 78 years old male and (**d**) a 93 years old female

Qualitative examination of all 24–2 HVF measurements showed no changes in the central visual field of all volunteers studied (Fig. 2, left row). In contrast, the HVF 60–4 measurements revealed a decreased nasal inferior threshold measurement beginning early in the 30 year old age group (Fig. 2a), and showed a continuous loss of retinal sensitivity with age (Fig. 2b-d). The inferior temporal quadrant retinal sensitivity was delayed and decreased in all age groups. However in the 90-year-old age group the peripheral visual field demonstrated markedly reduced retinal sensitivity in all four quadrants. The loss of retinal sensitivity started in the far periphery visual field (40 to 60 degrees nasal and 60 to 80 degrees temporal) and reached the outer rim of the central visual field (30 degrees) in the 10th decade of life. (To demonstrate more clinical information of the selected volunteers, the corresponding Heidelberg Retinal Tomography data and fundus photographies in Fig. 2 were attached as Additional file 4).

**Fig. 3:**
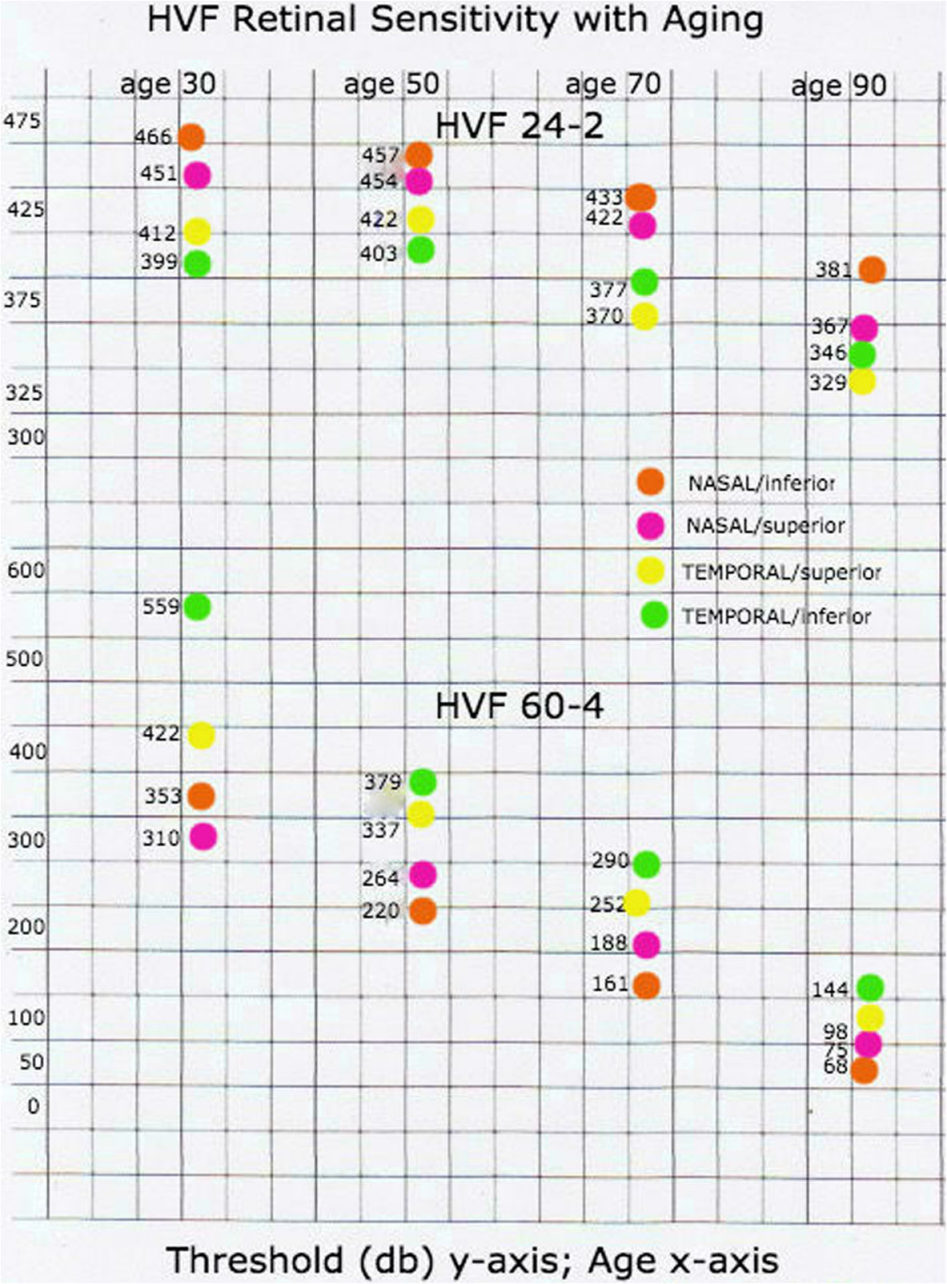
Graphic presentation of the mean threshold sum score levels of the four age groups. Note the decrease with age in all four quadrants (represented by a colour each) of the central (HVF 24–2) and peripheral (HVF 60–4) visual field

Quantitative measurements of the sum score of the visual field threshold revealed comparable numbers in all four quadrants of the HVF 24–2 (Table 1). These numbers showed an insignificant gradual decrease between each age group tested. However, comparing the extremes, the 30-year-old age group against the 90-year-old age group, all quadrants showed a sizable decrease of their retinal sensitivity scores (Fig. 3) which was statistically significant.

**Table 1:** Mean threshold levels and standard deviation (and median in parenthesis) of the Humphrey 24–2 visual field measurements in each quadrant (superior-nasal, superior-temporal, inferior-nasal, inferior-temporal) in four age groups (*n* = number of individuals tested)

Age decade	SupNas	SupTemp	InfNas	InfTemp
30ies (*n* = 5)	452 ± 23 (459)	412 ± 17 (410)	466 ± 20 (458)	399 ± 32 (408)
50ies (*n* = 5)	455 ± 8 (454)	415 ± 18 (411)	457 ± 11 (461)	403 ± 16 (403)
70ies (*n* = 5)	422 ± 13 (420)	370 ± 18 (378)	434 ± 19 (431)	378 ± 22 (389)
90ies (*n* = 5)	367 ± 29 (375)	329 ± 31 (322)	382 ± 22 (388)	346 ± 20 (340)

A statistical significant difference was only present comparing the individual quadrants mean threshold level of the 30ies age group and the 90ies

Quantitative evaluation of the HVF 60–4 revealed a dramatic and significant decrease of the retinal sensitivity scores in each quadrant between each age group (Table 2, Fig. 3). Using the 30 year old age group as a reference, the average sensitivity count decreased 14–27% in the 50-year-old age group, to 40–45% in the 70-year-old age group and to 73–78% in the 90-year-old age group. The inferior nasal quadrant showed the most dramatic decrease with remaining retinal sensitivity of 65% in the 50-year-old age group, 47% in the 70-year-old age group, and 20% in the 90-year-old age group.

**Table 2:** Mean threshold levels and standard deviation (and median in parenthesis) of the Humphrey 60–4 visual field collage (combination of ‘normal’ and ‘20 degrees nasal fixation measurements) in each quadrant (superior-nasal, superior-temporal, inferior-nasal, inferior-temporal) in four age groups (n = number of individuals tested)

Age decade	SupNas	SupTemp	InfNas	InfTemp
30ies (*n* = 5)	311 ± 51 (298)	426 ± 52 (435)	343 ± 38 (349)	520 ± 84 (537)
50ies (*n* = 5)	264 ± 38 (277)	319 ± 54 (318)	220 ± 71 (212)	379 ± 83 (369)
70ies (*n* = 5)	185 ± 33 (174)	252 ± 69 (228)	161 ± 70 (145)	290 ± 65 (301)
90ies (*n* = 5)	69 ± 44 (75)	98 ± 50 (108)	68 ± 20 (58)	144 ± 13 (146)

For quadrant comparison one has to keep in mind, that the superior HVF measures only to 50 degrees due to the paucity of retinal ganglion cells in the inferior peripheral retina. Therefore, only nasal and temporal quadrants can be compared in the superior and inferior half respectively. Each neighbouring age group showed a significant decrease of the individual quadrants mean threshold level in the older age group

## Discussion

Few publications exist about the peripheral 40–60 degree of the visual field but none covers the peripheral 60–80 degrees of the nasal retina [19–22]. In a number of publications the term peripheral visual field is a misnomer used for the peripheral part of the central visual field and not the real peripheral visual field beyond 30 degrees.

Our data presented in this paper support chronologic aging processes of the visual field as a marker for retinal ganglion cell function as suggested previously by several authors mentioned in the introduction. Corroborating earlier findings [23] we showed that the visual field changes predominantly affect the retinal periphery in normal healthy subjects. These changes continue over one’s life span (between the 4th and 10th decade of life). On the other hand we demonstrated that visual acuity in all volunteers remained unchanged, although a numeric decrease of the visual field threshold was observed in the older age groups. Certainly we are aware that this is a cross-sectional study, and therefore our conclusion has to be substantiated with more volunteers filling in the age gaps of our present sample and with longitudinal observations.

The aging factors involved in creating the different visual field results in the central and peripheral visual fields of the eye need further investigation. However, we would like to supplement our findings on inner retinal vascular supply described previously [18] and specifically indicate that the major factor for peripheral retinal ganglion cell apoptosis is insufficient retinal perfusion from the peripheral choriocapillaris and to a lesser degree the peripheral superficial retinal capillary bed.

While the choroid supplies the outer one third of the retina at the posterior pole measured by the central visual field it becomes more important for the nutrition of the inner retina in the region of the peripheral visual field. This is due to a reduction of the retinal thickness and the absence of the deep retinal capillary bed which terminates at 33 degrees temporal and 61 degrees nasal to the fovea. The morphology of the choriocapillaris, changes at the equator changes from a lobular to a fan-shaped configuration [24], present in the ora watershed zone.

Loss of endothelial cells and vascular irregularities of the choriocapillaris in this ora watershed zone were noted as early as in the 4th decade of life [25, 26]. However, Bruch’s membrane at the equator shows little change from the aging processes like collagen thickening [27] or increase of lipoprotein deposition [28–31]. This indicates a low exchange rate of substrates in the region of the equator. These known morphological changes might explain the increased vulnerability and therefore the earlier loss of retinal ganglion cells leading to the above peripheral visual field changes. Needless to say, more research is necessary to substantiate our findings.

Concerning ocular pathology, the described changes seem to be of specific importance when looking at ocular hypertension and early glaucomatous changes. This was recently demonstrated in a case study [32] and will be continued in more patients.

## Conclusions

In this small study on age-related changes of the peripheral and central HVF we found significant changes from the 4th decade of life onward affecting mainly the far periphery of the retina. Longitudinal observations are necessary to describe a linear versus a stepwise decline which was not possible in the presently used design. The data is a first base for differentiating normal aging versus pathological changes.

## Additional files

Additional file 1: Table S1. Cross sectional studies on age-related changes of the retinal nerve fiber layer thickness. Overview of the literature: clinical cross sectional studies describing thickness variations of the peripapillary retinal nerve fiber layer between healthy young and elderly subjects. (DOC 71 kb)

Additional file 2: Table S2. Individual testing time of the different Humphrey visual fields. The analysis shows the patient first initials, time the patient needed for their visual fields (VFs), their age and gender. FL - fixation loss, FP - false positive (“trigger happy”), FN - false negative (“falling asleep”). (DOC 105 kb)

Additiona file 3: Original single measurements of the HVF. All sum scores of the individual measurements in all four quadrants. (XLSX 11 kb)

Additional file 4: Heidelberg Retinal Tomography data and fundus photographies. Corresponding data to Fig. 2a-d. (ZIP 27125 kb)

## Abbreviation

HVF: Humphrey Visual Field
