# Supplementary material for: The peripheral and Central Humphrey visual field – morphological changes during aging
Source: BMC Ophthalmol. 2017 Jul 17;17:127. doi: 10.1186/s12886-017-0522-3 (PMC5514484; doi:10.1186/s12886-017-0522-3)
Supplement: Supplementary file 1 — Cross sectional studies on age-related changes of the retinal nerve fiber layer thickness. Overview of the literature: clinical cross sectional studies describing thickness variations of the peripapillary retinal nerve fiber layer between healthy young and elderly subjects. (DOC 71 kb) [file 12886_2017_522_MOESM1_ESM.doc]

Table 1. Cross sectional studies on age-related changes of the retinal nerve fiber layer thickness

| Study | Number of participants | Age range | Age classification | Regression coefficient |
| --- | --- | --- | --- | --- |
| Chauhan et al. 2015 | 246 | 19-87 | scatterplot | - 0.21µm/ year |
| Thapa et al. 2014 | 156 | 14-76 | decades | - 2.26µm/ decade |
| Patel et al. 2014 | 113 | 19-76 | scatterplot | - 0.206µm/ year |
| Appukuttan et al. 2014 | 105 | 20-75 | decade | - 1.57µm/ decade |
| Rao et al. 2014 | 73 | 19-68 | scatterplot | - 1µm/ decade |
| Celebi and Mirza 2013 | 121 | 20-59 | scatterplot | - 0.365µm/ year |
| Wang et al. 2013 | 1654 | 50-93 | scatterplot | - 0.5µm/ year |
| Khawaja et al. 2013 | 6309 | 48-90 | decade | - 1.53µm/ decade |
| Demirkaya et al. 2013 | 120 | 18-81 |  | Decrease with age |
| Mansoori et al. 2012 | 210 | ? | decade | - 0.116µm/ year |
| Leung et al. 2012 | 100 | 41-74 | scatterplot | - 0.33µm/ year |
| Feuer et al. 2011 | 425 | 18-85 | decades | - 2.4µm/ decade |
| Girkin et al. 2011 | 350 | 18-88 | scatterplot | - 0.18mm/ year |
| Kim et al. 2011 | 182 | 22-84 | scatterplot, decades | - 1.59µm/ decade |
| Mwanza et al. 2011 | 284 | 18-85 | scatterplot |  |
| Sung et al. 2009 | 124 | 18-85 | decades | - 0.45µm/ year |
| Harwerth and Wheat 2008 | ? | 25-95 | decades | ? |
| Parikh et al. 2007 | 187 | 5-75 | scatterplot | - 0.16µm/ year |
| Budenz et al. 2007 | 328 | 18-85 | csatterplot | - 2.0µm/ decade |
| Da Pozzo et al. 2006 | 324 | 21-85 | decades | - 0.08µm bis – 0.16µm/ year |
| Yamada et al. 2006 | 100 | ? | ? | Negative correlation |
| Sony et al. 2004 | 146 | 20-70 | 3 groups: <30 y, 31-50 y, >50 y | Only superior and inferior significant decrease |
| Alamouti and Funk 2003 | 100 | 6-79 | scatterplot | - 0.53µm/ year |
| Kanamori et al. 2003 | 144 | 16-84 | scatterplot | - 0.17%/ year |
| Varma et al. 2003 | 312 | 40-79 | ? | Thinner in older |
| Bowd et al. 2002 | 155 | 23-80 | 3 groups: 23-43 y, 44-64 y, >64 y | Weak negative correlation |
| García Feijoó and García Sánchez 2001 | 48 | ? | ? | Decrease with age |
| Lee and Mok 2000 | 159 | 10-78 | decades | - 1.9µm/ decade |
| Toprak and Yilmaz 2000 | 38 | 20-78 | scatterplot | Significant decrease with age |

Literature to Table 1:

Chauhan BC, Danthurebandara VM, Sharpe GP, Demirel S, Girkin CA, Mardin CY, Scheuerle AF, Burgoyne CF. Bruch's Membrane Opening Minimum Rim Width and Retinal Nerve Fiber Layer Thickness in a Normal White Population: A Multicenter Study. Ophthalmology. 2015 Sep;122(9):1786-94.

Thapa M, Khanal S, Shrestha GB, Sharma AK. Retinal nerve fibre layer thickness in a healthy Nepalese population by spectral domain optical coherence tomography. Nepal J Ophthalmol. 2014 Jul-Dec;6(2):131-9.

Patel NB, Lim M, Gajjar A, Evans KB, Harwerth RS. Age-associated changes in the retinal nerve fiber layer and optic nerve head. Invest Ophthalmol Vis Sci. 2014 Jul 22;55(8):5134-43.

Appukuttan B, Giridhar A, Gopalakrishnan M, Sivaprasad S. Normative spectral domain optical coherence tomography data on macular and retinal nerve fiber layer thickness in Indians. Indian J Ophthalmol. 2014 Mar;62(3):316-21.

Rao HL, Venkatesh CR, Vidyasagar K, Yadav RK, Addepalli UK, Jude A, Senthil S, Garudadri CS. Retinal nerve fiber layer measurements by scanning laser polarimetry with enhanced corneal compensation in healthy subjects. J Glaucoma. 2014 Dec;23(9):589-93.

Celebi AR, Mirza GE. Age-related change in retinal nerve fiber layer thickness measured with spectral domain optical coherence tomography. Invest Ophthalmol Vis Sci. 2013 Dec 11;54(13):8095-103.

Wang YX, Pan Z, Zhao L, You QS, Xu L, Jonas JB. Retinal nerve fiber layer thickness. The Beijing Eye Study 2011. PLoS One. 2013 Jun 24;8(6):e66763.

Khawaja AP, Chan MP, Garway-Heath DF, Broadway DC, Luben R, Sherwin JC, Hayat S, Khaw KT, Foster PJ. Associations with retinal nerve fiber layer measures in the EPIC-Norfolk Eye Study. Invest Ophthalmol Vis Sci. 2013 Jul 26;54(7):5028-34.

Demirkaya N, van Dijk HW, van Schuppen SM, Abràmoff MD, Garvin MK, Sonka M, Schlingemann RO, Verbraak FD. Effect of age on individual retinal layer thickness in normal eyes as measured with spectral-domain optical coherence tomography. Invest Ophthalmol Vis Sci. 2013 Jul 22;54(7):4934-40.

Mansoori T, Viswanath K, Balakrishna N. Quantification of retinal nerve fiber layer thickness using spectral domain optical coherence tomography in normal Indian population. Indian J Ophthalmol. 2012 Nov-Dec;60(6):555-8.

Leung CK, Yu M, Weinreb RN, Ye C, Liu S, Lai G, Lam DS. Retinal nerve fiber layer imaging with spectral-domain optical coherence tomography: a prospective analysis of age-related loss. Ophthalmology. 2012 Apr;119(4):731-7.

Feuer WJ, Budenz DL, Anderson DR, Cantor L, Greenfield DS, Savell J, Schuman JS, Varma R. Topographic differences in the age-related changes in the retinal nerve fiber layer of normal eyes measured by Stratus optical coherence tomography. J Glaucoma. 2011 Mar;20(3):133-8.

Girkin CA, McGwin G Jr, Sinai MJ, Sekhar GC, Fingeret M, Wollstein G, Varma R, Greenfield D, Liebmann J, Araie M, Tomita G, Maeda N, Garway-Heath DF. Variation in optic nerve and macular structure with age and race with spectral-domain optical coherence tomography. Ophthalmology. 2011 Dec;118(12):2403-8.

Kim NR, Kim JH, Lee J, Lee ES, Seong GJ, Kim CY. Determinants of perimacular inner retinal layer thickness in normal eyes measured by Fourier-domain optical coherence tomography. Invest Ophthalmol Vis Sci. 2011 May 18;52(6):3413-8.

Sung KR, Wollstein G, Bilonick RA, Townsend KA, Ishikawa H, Kagemann L, Noecker RJ, Fujimoto JG, Schuman JS. Effects of age on optical coherence tomography measurements of healthy retinal nerve fiber layer, macula, and optic nerve head. Ophthalmology. 2009 Jun;116(6):1119-24.

Harwerth RS, Wheat JL. Modeling the effects of aging on retinal ganglion cell density and nerve fiber layer thickness. Graefes Arch Clin Exp Ophthalmol. 2008 Feb;246(2):305-14.

Budenz DL, Anderson DR, Varma R, Schuman J, Cantor L, Savell J, Greenfield DS, Patella VM, Quigley HA, Tielsch J. Determinants of normal retinal nerve fiber layer thickness measured by Stratus OCT. Ophthalmology. 2007 Jun;114(6):1046-52.

Da Pozzo S, Iacono P, Marchesan R, Minutola D, Ravalico G. The effect of ageing on retinal nerve fibre layer thickness: an evaluation by scanning laser polarimetry with variable corneal compensation. Acta Ophthalmol Scand. 2006 Jun;84(3):375-9.

Yamada H, Yamakawa Y, Chiba M, Wakakura M. Evaluation of the effect of aging on retinal nerve fiber thickness of normal Japanese measured by optical coherence tomography. Nihon Ganka Gakkai Zasshi. 2006 Mar;110(3):165-70.

Sony P, Sihota R, Tewari HK, Venkatesh P, Singh R. Quantification of the retinal nerve fibre layer thickness in normal Indian eyes with optical coherence tomography. Indian J Ophthalmol. 2004 Dec;52(4):303-9.

Alamouti B, Funk J. Retinal thickness decreases with age: an OCT study. Br J Ophthalmol. 2003 Jul;87(7):899-901.

Kanamori A, Escano MF, Eno A, Nakamura M, Maeda H, Seya R, Ishibashi K, Negi A. Evaluation of the effect of aging on retinal nerve fiber layer thickness measured by optical coherence tomography. Ophthalmologica. 2003 Jul-Aug;217(4):273-8.

Varma R, Bazzaz S, Lai M. Optical tomography-measured retinal nerve fiber layer thickness in normal latinos. Invest Ophthalmol Vis Sci. 2003 Aug;44(8):3369-73.

Bowd C, Zangwill LM, Blumenthal EZ, Vasile C, Boehm AG, Gokhale PA, Mohammadi K, Amini P, Sankary TM, Weinreb RN. Imaging of the optic disc and retinal nerve fiber layer: the effects of age, optic disc area, refractive error, and gender. J Opt Soc Am A Opt Image Sci Vis. 2002 Jan;19(1):197-207.

García Feijoó J, García Sánchez J. Analysis of aged-related nerve fiber layer thickness changes with laser polarimetry. Arch Soc Esp Oftalmol. 2001 Aug;76(8):477-83.

Lee VW, Mok KH. Nerve fibre layer measurement of the Hong Kong Chinese population by scanning laser polarimetry. Eye (Lond). 2000 Jun;14 ( Pt 3A):371-4.

Toprak AB, Yilmaz OF. Relation of optic disc topography and age to thickness of retinal nerve fibre layer as measured using scanning laser polarimetry, in normal subjects. Br J Ophthalmol. 2000 May;84(5):473-8.
