# Supplementary material for: The peripheral and Central Humphrey visual field – morphological changes during aging
Source: BMC Ophthalmol. 2017 Jul 17;17:127. doi: 10.1186/s12886-017-0522-3 (PMC5514484; doi:10.1186/s12886-017-0522-3)
Supplement: Supplementary file 2 — Individual testing time of the different Humphrey visual fields. The analysis shows the patient first initials, time the patient needed for their visual fields (VFs), their age and gender. FL - fixation loss, FP - false positive (“trigger happy”), FN - false negative (“falling asleep”). (DOC 105 kb) [file 12886_2017_522_MOESM2_ESM.doc]

Table 2. Individual testing time of the different Humphrey visual fields. The analysis shows the patient first initials, time the patient needed for their VFs, their age and gender.

FL - fixation loss

FP - false positive (“trigger happy”)

FN - false negative (“falling asleep”)

| AGE 90’S |  |  |  |
| --- | --- | --- | --- |
| **DR - FEMALE AGE 97** | **24 - 2** | **60 - 4** | **60 – 4 DOT** |
| FL | 10 | 1 | 0 |
| FP (+) | 5 | 1 | 1 |
| FN (-) | 4 | 45 | 10 |
| TIME | 5:58 | 9:21 | 7:31 |
| **JH - FEMALE AGE 93** |  |  |  |
|  | 0 | 0 | 0 |
|  | 1 | 1 | 1 |
|  | 2 | 25 | 0 |
|  | 6:09 | 8:28 | 8:12 |
| **AM – MALE AGE 90** |  |  |  |
|  | 1 | 4 | 16 |
|  | 3 | 2 | 0 |
|  | 0 | 8 | 0 |
|  | 5:55 | 7:54 | 8:00 |
| **SB – MALE AGE 90** |  |  |  |
|  | 0 | 0 | 1 |
|  | 2 | 0 | 0 |
|  | 0 | 7 | 9 |
|  | 4:41 | 8:01 | 7:21 |
| **EDC – FEMALE AGE 92** |  |  |  |
|  | 4 | 0 | 18 |
|  | 1 | 0 | 3 |
|  | 3 | 0 | 7 |
|  | 5:25 | 7:04 | 8:44 |

| AGE 70’S |  |  |  |
| --- | --- | --- | --- |
| **PR - MALE AGE 78** | **24 - 2** | **60 - 4** | **60 – 4 DOT** |
| FL | 1 | 1 | 0 |
| FP (+) | 2 | 0 | 2 |
| FN (-) | 0 | 7 | 0 |
| TIME | 4:44 | 7:56 | 6:35 |
| **SB – MALE AGE 73** |  |  |  |
|  | 2 | 1 | 17 |
|  | 4 | 0 | 0 |
|  | 4 | 0 | 15 |
|  | 4:57 | 7:18 | 8:25 |
| **SC – MALE AGE 72** |  |  |  |
|  | 0 | 0 | 16 |
|  | 1 | 2 | 1 |
|  | 0 | 25 | 16 |
|  | 5:01 | 6:53 | 8:13 |
| **CC - FEMALE AGE 72** |  |  |  |
|  | 0 | 0 | 0 |
|  | 0 | 0 | 0 |
|  | 0 | 15 | 0 |
|  | 4:49 | 7:37 | 7:24 |
| **BB - FEMALE AGE 71** |  |  |  |
|  | 1 | 2 | 19 |
|  | 1 | 0 | 2 |
|  | 0 | 0 | 14 |
|  | 5:16 | 8:27 | 8:53 |

| AGE 50’S |  |  |  |
| --- | --- | --- | --- |
| **DP – MALE AGE 56** | **24 - 2** | **60 - 4** | **60 – 4 DOT** |
| FL | 0 | 0 | 0 |
| FP (+) | 6 | 2 | 1 |
| FN (-) | 0 | 0 | 6 |
| TIME | 4:26 | 6:32 | 6:19 |
| **SR - FEMALE AGE 57** |  |  |  |
|  | 4 | 2 | 0 |
|  | 6 | 3 | 2 |
|  | 0 | 0 | 0 |
|  | 4:47 | 8:27 | 7:19 |
| **LB – FEMALE AGE 53** |  |  |  |
|  | 3 | 0 | 0 |
|  | 5 | 0 | 0 |
|  | 0 | 0 | 8 |
|  | 4:47 | 6:21 | 6:22 |
| **MS - FEMALE AGE 52** |  |  |  |
|  | 0 | 7 | 17 |
|  | 1 | 0 | 2 |
|  | 0 | 0 | 0 |
|  | 4:02 | 8:13 | 8:18 |
| **MP – FEMALE AGE 56** |  |  |  |
|  | 4 | 1 | 0 |
|  | 6 | 11 | 9 |
|  | 0 | 7 | 8 |
|  | 4:37 | 8:07 | 7:05 |

| AGE 30’S |  |  |  |
| --- | --- | --- | --- |
| **JK – MALE AGE 30** | **24 - 2** | **60 - 4** | **60 – 4 DOT** |
| FL | 0 | 1 | 1 |
| FP (+) | 0 | 7 | 1 |
| FN (-) | 0 | 0 | 0 |
| TIME | 4:50 | 6:03 | 5:34 |
| **SM – FEMALE AGE 38** | 2 | 18 | 8 |
|  | 3 | 2 | 7 |
|  | 0 | 14 | 2 |
|  | 5:33 | 6:58 | 6:32 |
| **TM – MALE AGE 33** | 1 | 0 | 0 |
|  | 7 | 6 | 0 |
|  | 0 | 0 | 0 |
|  | 4:18 | 6:27 | 5:32 |
| **JA – FEMALE AGE 31** |  |  |  |
|  | 1 | 0 | 2 |
|  | 8 | 3 | 6 |
|  | 0 | 0 | 0 |
|  | 4:34 | 6:04 | 6:52 |
| **JM - FEMALE AGE 31** |  |  |  |
|  | 0 | 0 | 14 |
|  | 0 | 3 | 3 |
|  | 0 | 0 | 0 |
|  | 4:18 | 5:17 | 5:38 |
