# Supplementary figures and images for: The peripheral and Central Humphrey visual field – morphological changes during aging
Source: BMC Ophthalmol. 2017 Jul 17;17:127. doi: 10.1186/s12886-017-0522-3 (PMC5514484; doi:10.1186/s12886-017-0522-3)

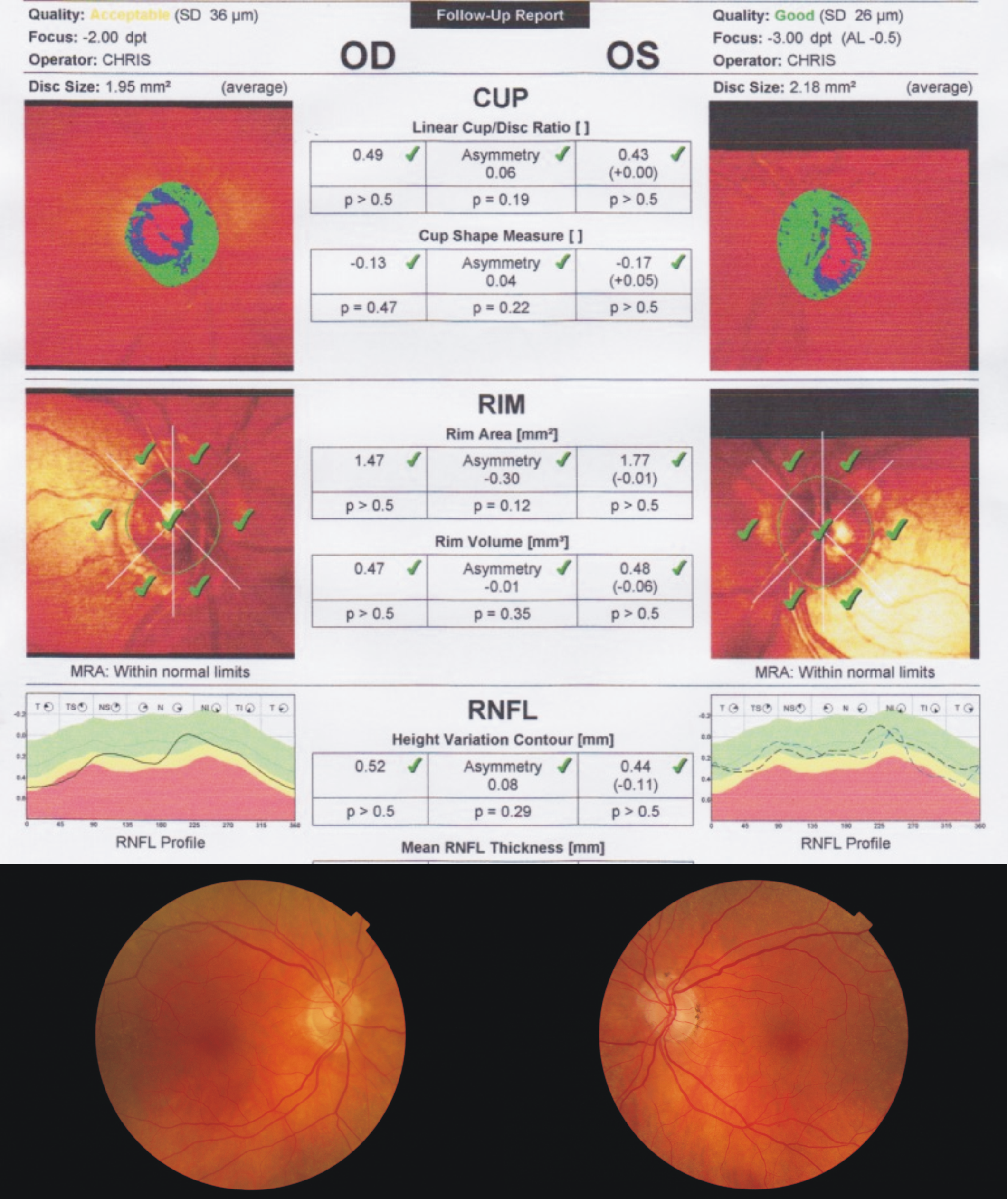

Supplement: Supplementary file 4 — Heidelberg Retinal Tomography data and fundus photographies. Corresponding data to Fig. 2a-d. (ZIP 27125 kb) [file 12886_2017_522_MOESM4_ESM.zip › 90er-ergR2.tif]

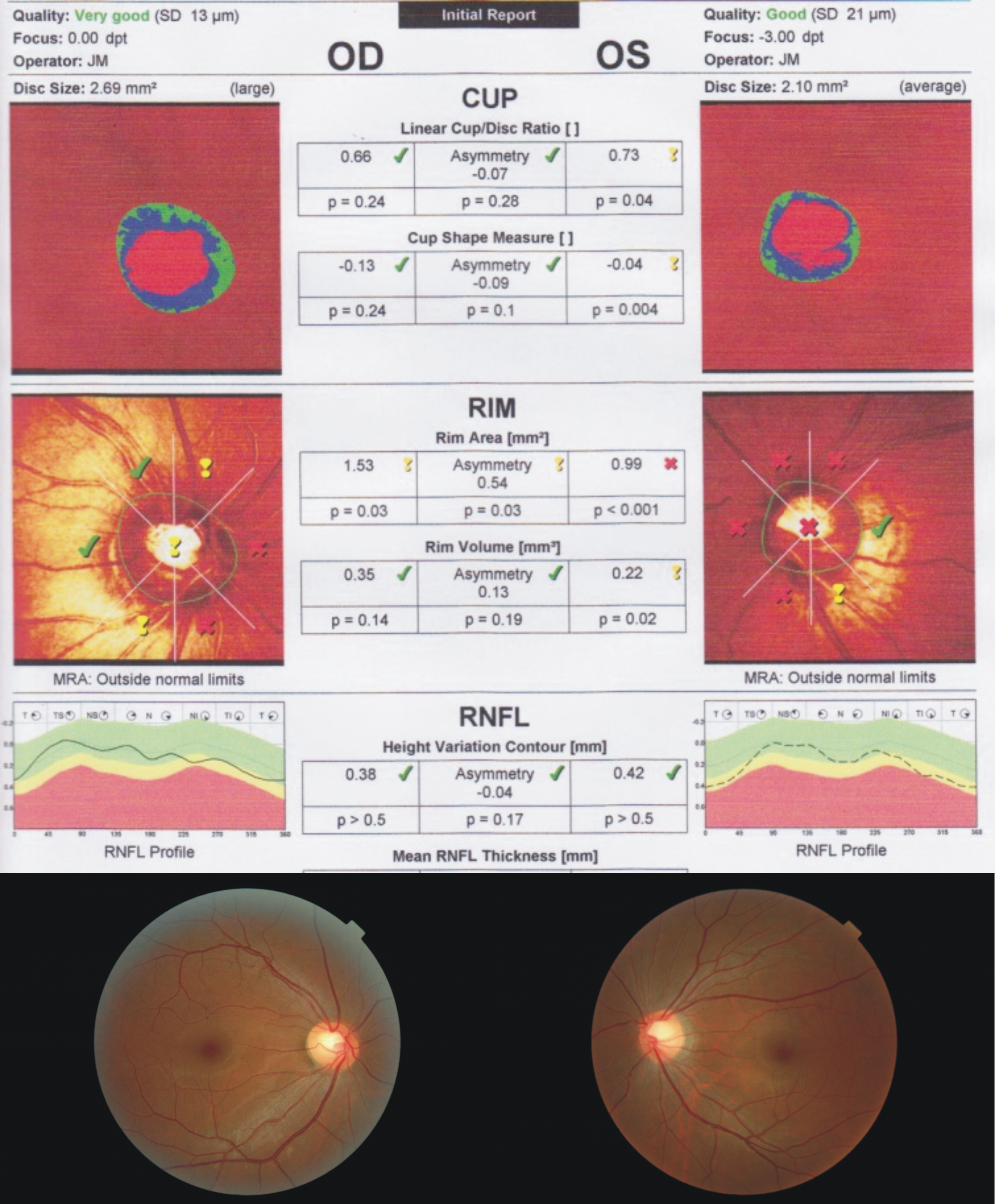

Supplement: Supplementary file 4 — Heidelberg Retinal Tomography data and fundus photographies. Corresponding data to Fig. 2a-d. (ZIP 27125 kb) [file 12886_2017_522_MOESM4_ESM.zip › 30er-ergR2.TIF]

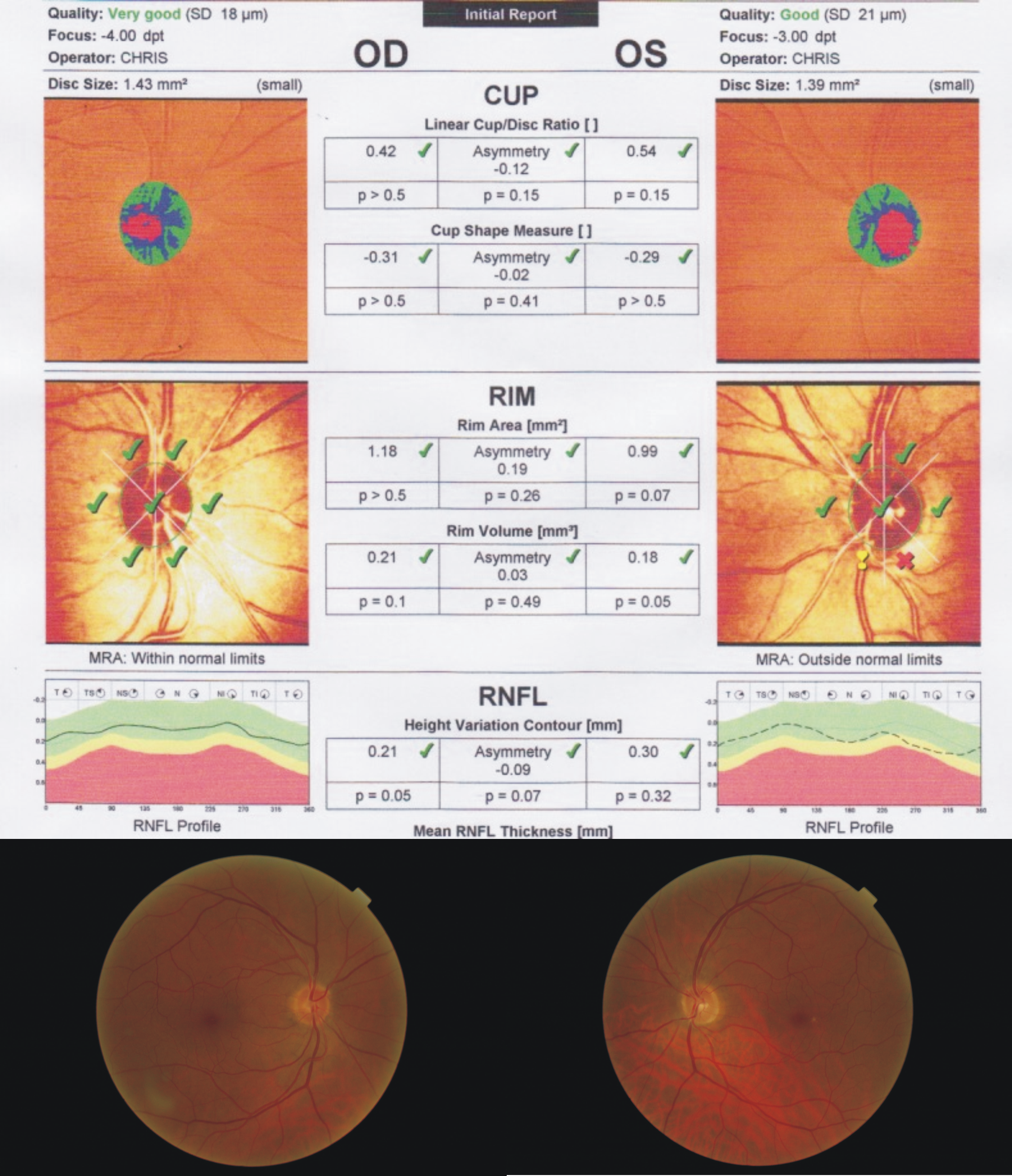

Supplement: Supplementary file 4 — Heidelberg Retinal Tomography data and fundus photographies. Corresponding data to Fig. 2a-d. (ZIP 27125 kb) [file 12886_2017_522_MOESM4_ESM.zip › 50er-ergR2.tif]

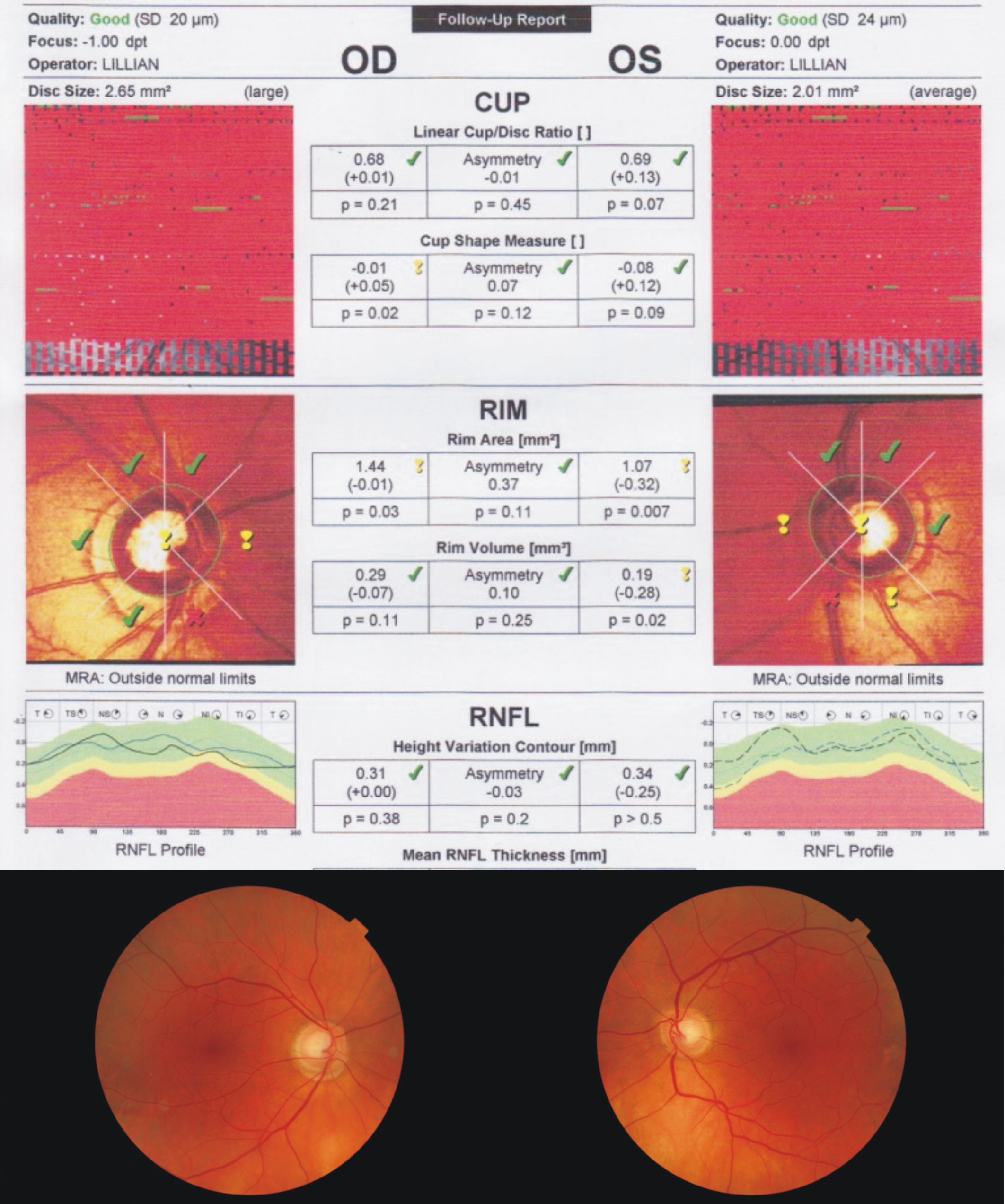

Supplement: Supplementary file 4 — Heidelberg Retinal Tomography data and fundus photographies. Corresponding data to Fig. 2a-d. (ZIP 27125 kb) [file 12886_2017_522_MOESM4_ESM.zip › 70er-ergR2.tif]
